# Optimization of a CRISPR-Cas9 *in vitro* protocol for targeting the SIX9 gene of *Fusarium oxysporum* f.sp. *cubense* race 1 associated with banana Fusarium wilt

**DOI:** 10.3389/fpls.2025.1523884

**Published:** 2025-03-10

**Authors:** Liliana Villao, Jeffrey Vargas, Nardy Diez, Freddy Magdama, Efrén Santos-Ordóñez

**Affiliations:** ^1^ Centro de Investigaciones Biotecnológicas del Ecuador, ESPOL Polytechnic University, Escuela Superior Politécnica del Litoral (ESPOL), Guayaquil, Ecuador; ^2^ Facultad de Ciencias de la Vida, ESPOL Polytechnic University, Escuela Superior Politécnica del Litoral (ESPOL), Guayaquil, Ecuador

**Keywords:** Fusarium wilt disease, gene editing, CRISPR Cas9, SIX proteins, *Musa* spp.

## Abstract

**Introduction:**

Fusarium wilt of bananas (*Musa* spp.), a threat to sustainable banana production worldwide, necessitates immediate action to control the disease. The current strategies are centered on preventing its spread or developing resistant varieties. However, very little is known about the genetic machinery used by the fungus to infect and kill banana plants. Therefore, research should the focused also in understanding the plant-pathogen molecular interaction by targeting virulent genes for knock-out in Fusarium. This study aims to standardize a gene editing protocol using CRISPR Cas9 technology in *Fusarium oxysporum* f.sp. *cubense* race 1 (Foc1); specifically, to induce targeted mutations on a particular effector gene, SIX9, of Foc1.

**Methods:**

An *in vitro* protocol was optimized for the production of the Cas9 protein to target the SIX9 gene testing two gRNAs, by expression and purification of the Cas9, included in plasmids pHis-parallel1 and pMJ922, in *E. coli* BL21 Rosetta, independently.

**Results:**

Results demonstrated that the produced Cas9 exhibits high enzymatic activity, comparable to the commercial standard. These findings underscore the robustness of the in-house enzyme and highlight its suitability for future research and biotechnological applications.

**Discussion:**

This protocol facilitates the production of recombinant Cas9, enabling its use in various experimental settings and accelerating research in targeted gene editing, an area of significant relevance today. This protocol will support future studies on banana-Fusarium interaction by identifying candidate genes for disease resistance for the plant, or lack of virulence for the pathogen, by establishing the function of SIX effector proteins and evaluating the fungus’s infection capacity through pathogenicity assays.

## 1 Introduction

### 1.1 General aspects of bananas (*Musa* spp.)

Bananas (*Musa* spp.) are cultivated in over 135 countries in tropical and subtropical regions and are considered one of the most important crops in the world. They represent a significant source of calories for over 500 million people (FAO, 2023). They generate income for millions of small farmers and represent a key export product for many countries (Evizal and Prasmatiwi, 2024). Banana is affected by several biotic stress, including black leaf streak and Fusarium wilt. A key step to manage disease by biotechnological tools is the understanding of plant-pathogen interactions at the molecular level, in which virulent genes from the pathogen are identified and could become the target to control disease. Therefore, the implementation of a methodology to introduce mutations to virulent genes is relevant. In this scenario, the CRISPR-Cas9 is an important tool to determinate gene function and identify virulent genes in pathogens.

From a nutrition perspective, bananas are highly nutritious fruits with a wide range of health benefits due to their rich composition of vitamins, minerals, and bioactive compounds. Their high potassium content is essential for cardiovascular health and muscle function. This fruit is rich in vitamins B6, C, and potassium—key nutrients for metabolism and immune function. Additionally, their dietary fiber content, both soluble and insoluble, plays a crucial role in promoting digestive health. (Kumari et al., 2023)

Bananas have great versatility that goes beyond their consumption as fresh fruit. They are increasingly used in producing food products, such as banana flour, which is gluten-free and rich in resistant starch, offering additional health benefits (Keeratiburana et al., 2024). In addition, banana by-products, such as peels and pseudo-stems, are being researched for their nutritional and functional properties, which help reduce waste and promote sustainable farming practices (Kumari et al., 2023). Their wide range of applications in food products and their health benefits highlight their importance as a fundamental crop.

### 1.2 Fusarium oxysporum causal agent

The disease Fusarium wilt, caused by the fungus *Fusarium oxysporum* f. sp. *cubense* (Foc), demands immediate attention and action. This disease poses a significant risk to the current banana industry, and urgent measures are needed to prevent its further spread. By 2040, *Fusarium oxysporum* f. sp. *cubense* tropical race 4 (Foc TR4), responsible for Fusarium wilt in bananas, will affect approximately 17% of the global banana-cultivated areas. This would represent a loss of 36 million tons of production, valued at over 10 billion USD (Staver et al., 2020).

In 2022, Latin America was responsible for 60% of the 21 million tons of bananas exported worldwide. Eight countries from Latin America and the Caribbean (Ecuador, Colombia, Costa Rica, Guatemala, Honduras, Panama, Mexico, and the Dominican Republic) are among the top ten global exporters. The top three organic banana exporters are Ecuador, the Dominican Republic, and Peru (Loeillet, 2023).

In previous years, and despite attempts to control the spread of race 1 of this fungus, it was impossible to sustain production using a susceptible variety, replacing the ‘Gros Michel’ (genotype AAA) cultivar with Cavendish cultivars, including ‘Grand Naine’ and ‘Williams’ (genotypes AAA), now representing an estimated 40% of the global supply (Bragard et al., 2022; Martínez et al., 2023; Martínez-Solórzano et al., 2020)

Unlike Foc1, this new race affects not only the Cavendish cultivar but also 60% of all banana varieties worldwide. Foc TR4 has spread to more than 20 countries, including Taiwan, Indonesia, Malaysia, Australia, India, England, Colombia, Peru, and Venezuela (Dita et al., 2018; FAO, 2023; Martínez et al., 2023). This severely threatens Ecuador, the world’s largest banana exporter, with a 35% international market share.

Despite containment efforts, the spread of the fungus. The lack of genetic diversity, intensive agricultural practices, insufficient awareness, and inadequate public policies exacerbate the issue despite attempts to implement biosafety measures. Fusarium wilt poses a significant threat to the food security of millions of people.

*Fusarium oxysporum* is a Species Complex (FOSC) consisting of pathogenic and non-pathogenic races. It is a cosmopolitan fungus found in various ecological niches, colonizing plant roots or as a saprophyte in the soil (González et al., 2012). Those races causing disease in specific crops have been classified into special forms (*forma* sp*ecialis*). More than 120 f.sp. of *F. oxysporum* have been reported, including *F. oxysporum* f.sp. cubense (Foc), for its specificity in exclusively attacking bananas (López-Zapata and Castaño-Zapata, 2019).

Three pathogen races are identified based on their ability to cause disease in specific banana cultivars. Race 1 affects banana varieties such as ‘Gros Michel’ (AAA), ‘Manzano’ (AAB), and ‘Pisang Awak.’ Race 2 affects cooking bananas, especially those of the ‘Bluggoe’ (ABB) subgroup. Race 3, which affects Heliconia species, tropical relatives of bananas, is mentioned. Foc Race 3 is not considered part of *Fusarium oxysporum* f. sp. cubense (Foc; Ploetz, 2015).

Race 4 affects all varieties susceptible to races 1 and 2, with a particular impact on cultivars of the Cavendish subgroup. Foc race 4 is subdivided into Subtropical Race 4 (SR4) and Tropical Race 4 (TR4). SR4 causes disease in subtropical areas, especially under stress conditions such as drought and low temperatures. Although Cavendish bananas are not affected by SR4 in tropical regions, SR4 strains are distributed and affect ‘Gros Michel’ in tropical areas, classified as R1 (Munhoz et al., 2024). In contrast, TR4 causes disease in tropical regions, even without stress factors (Dita et al., 2018; Magdama et al., 2019).

FocTR4 is a quarantine pathogen due to its destructive capacity and complex management (Magdama et al., 2020). FocTR4 spread typically occurs through the movement of contaminated soil, infected plant material, tools, contaminated farm equipment, shoes, and other means (Izquierdo-García et al., 2021). Runoff and contaminated river water used for irrigation also contribute to the fungus’s dispersion. (Ordonez et al., 2015).

The progression of this pathogen causes vascular obstruction, affecting water translocation and physiological processes such as photosynthesis. The most common symptoms associated with *Fusarium* wilt include yellowing, wilting, cracking of the pseudostem, and internal necrosis with reddish-brown discoloration (López-Zapata and Castaño-Zapata, 2019).

Foc is a complex pathogen, and its disease is challenging to manage. Due to its saprophytic characteristics and the ability to produce resistant structures called chlamydospores, its eradication is nearly impossible. According to reports, chlamydospores can survive in the soil for over 20 years (Buddenhagen, 2009).

Currently, banana producers’ biosecurity measures in the face of a possible incursion of Foc are short and medium-term strategies focused on prevention (O’Neill et al., 2016). However, given the ongoing spread of the pathogen and the industry’s high dependence on Cavendish-type varieties, which are susceptible to FocTR4, developing and adopting new resistant or tolerant varieties to the disease is imperative.

### 1.3 Action of Secreted in Xylem proteins

Foc is polyphyletic, and there is limited knowledge about the genetic basis of its specificity to bananas. Recent evidence shows that *F. oxysporum* has evolved complex mechanisms to overcome plant immunity by secreting proteins called effectors inside the plant cell (Jangir et al., 2021; Michielse and Rep, 2009; Redkar et al., 2022). Effectors are proteins or other molecules (e.g., secondary metabolites and small RNAs) linked to host processes that facilitate pathogen colonization and disease advancement (Carvalhais et al., 2019; de Sain and Rep, 2015).

Researchers initially identified these effectors in the sap proteome of tomato plants infected with the special form *F. oxysporum* f.sp. *lycopersici*, naming them SIX genes for Secreted in Xylem (Gawehns et al., 2015; Houterman et al., 2007). So far, 15 proteins related to the SIX genes have been identified: *F. oxysporum* formae specialis (An et al., 2019; Jobe et al., 2024; Yu et al., 2024). Studies have shown that these effectors are located in specific regions of the fungal ‘accessory genome’ composed of mobile chromosomes (Rep et al., 2004). Despite their mobility, these chromosomes play an important role under specific conditions, conferring pathogenicity against particular species.

One of the most well-documented examples is the pathogenic chromosome of *F. oxysporum* f.sp. *lycopersici*, where its absence does not affect the strain’s normal development but does affect its ability to infect the tomato plant (Lievens et al., 2009). Indeed, recent studies have shown that strains from different lineages within the same *formae* sp*eciales* contain identical SIX gene sequences. Despite their significant genetic differentiation, the presence of these homologous genes unifies them in their ability to cause disease, suggesting the hypothesis of horizontal transfer of genetic material (Czislowski et al., 2018). The SIX9 gene has been identified in FocTR4 (Maymon et al., 2020) but is also present in Foc1 (An et al., 2019).

Furthermore, previous research has shown that the SIX1 and SIX9 genes are present in all reported genotypes, including Foc1, Foc2, FocSTR4 and FocTR4 (Czislowski et al., 2018). The SIX9 gene is found in multiple copies in the tropical race 4, and its expression increases upon contact with the host (banana), suggesting that the product of this gene needs to accumulate at high levels within a short period (An et al., 2019; Maymon et al., 2020). However, understanding the genetic basis of what makes Foc fully pathogenic towards bananas is inconclusive. Therefore, gene functional analyses of the SIX genes should be performed to have a better insight in this plant-pathogen interaction.

### 1.4 Advances in genetic modification: The CRISPR-Cas9 revolution

Several years ago, the gene-editing technology known as CRISPR-Cas9 (an acronym for Clustered Regularly Interspaced Short Palindromic Repeats) was developed (reviewed by Huang et al., 2022). This technology utilizes the adaptive immune systems of various microorganisms to make specific and targeted cuts in DNA (Wei and Li, 2023). It has demonstrated high compatibility with most prokaryotic or eukaryotic species (Wu et al., 2014). One of the main requirements for this system to function is the presence of a small sequence (for *Streptococcus pyogenes*, it is 5´-NGG-3´) known as the protospacer adjacent motif (PAM), which is complementary to the site of the DNA under study and allows the cell to distinguish between its DNA and foreign DNA during the expression stage (Marraffini, 2016).

The Cas9 protein exhibits some variations, one of the most interesting being fused with a recombinant nuclear localization signal (NLS) at the C and N terminals, which enables precise DNA cleavage, ultimately forming a ribonucleoprotein complex (RNP) along with the guide RNA (Lino et al., 2018; Zhang et al., 2020). Genome editing based on ribonucleoprotein complexes provides rapid and efficient genome editing. Once transfection is completed, endogenous proteases within cells rapidly degrade the genome (Gong et al., 2021). Unlike other systems, the RNP does not require translation or transcription processes, thus avoiding the risk of inserting foreign DNA into the genome (Zhang et al., 2020). Additionally, it has been demonstrated that RNPs cut chromosomal target sites immediately after transfection and are rapidly degraded by endogenous proteases in cells. This helps reduce the effects of mosaicism, off-targets, or any genetic variation in regenerated plants (Bhattacharjee et al., 2023; Woo et al., 2015).

The assembly of RNPs only requires incubation periods, during which the Cas protein is mixed with the synthesized gRNA (Zhang et al., 2020). However, this technique still faces many technological challenges and variations in different experimental protocols, including the Cas::gRNA ratio. On multiple occasions, adding higher concentrations of Cas does not necessarily improve efficiency (Feng et al., 2022). Additionally, several methods for RNP delivery exist, such as using protoplasts, *Agrobacterium tumefaciens*, or biolistics, each with its advantages and disadvantages (Laforest and Nadakuduti, 2022; Nadakuduti and Enciso-Rodríguez, 2021; Sandhya et al., 2020). Protoplasts have been one of the most commonly used methods as they facilitate direct entry into the cell and allow the generation of whole plants from edited cells, although they have a low regeneration percentage in certain species.

On the other hand, *Agrobacterium* offers greater stability, which is crucial for long-term expression (Peterson et al., 2021). However, it can present challenges, such as random integration into the plant genome, which could lead to undesired effects. Additionally, eliminating these DNA sequences from the transformed plant or any other foreign DNA is often not feasible or difficult in asexually reproducing plant species. (Douches et al., 2020; Villao-Uzho et al., 2023).

Considering these cases where undesired results can occur, it is important to note the significance of understanding the functions of essential genes for plant cellular functions, including replication and cellular repair (Meiliana et al., 2017). The sustainable production of bananas faces significant challenges due to diseases such as Fusarium Wilt, which threaten the viability of this essential crop worldwide. Given the limitations of conventional breeding, such as the high ploidy and low fertility of bananas, new breeding techniques (NBTs), particularly gene editing through CRISPR/Cas9, emerge as a biotechnological tool for developing resistant varieties and ensuring the continuity of this key resource.

The integration of innovative approaches, such as the functional study of essential genes related to critical cellular processes and the deactivation of pathogenicity genes in *Fusarium oxysporum* f. sp. *cubense* (Foc), not only broadens our understanding of the underlying molecular mechanisms but also opens new perspectives for effectively addressing one of the greatest phytosanitary challenges in banana cultivation.

In this context, after producing the Cas9 protein, we evaluated its effectiveness compared to a commercial Cas9. This comparison aimed to determine whether our protocol could generate a Cas9 protein with comparable or superior performance to the commercially available options, resulting in a more efficient and accessible tool for gene editing in bananas and other crops.

This study contributes to the advancement of genetic improvement of this crop, laying a solid foundation for implementing sustainable and effective strategies against Fusarium wilt. Furthermore, the identification and deactivation of genes responsible for pathogenicity in *Fusarium oxysporum* f. sp. *cubense* using tools like CRISPR/Cas9 could significantly enhance the genetic improvement efforts for bananas by directly interfering with the pathogen-plant interaction and boosting the resistance of cultivated varieties to this devastating disease.

## 2 Materials and methods

### 2.1 Isolation of *Fusarium oxysporum* race 1 strains

For the editing assays, strains EC44-M-GM1 and O-1968 were used, characterized as Foc race 1 (Foc1), and preserved in the Phytopathology Laboratory of the Biotechnological Research Center of Ecuador (CIBE; Magdama et al., 2020).

### 2.2 In *silico* design and sgRNA synthesis

For the design of gRNAs, the Breaking-Cas software was utilized (Oliveros et al., 2016). Various factors should be considered to avoid cuts in other *loci* and potential off-target effects, including the protospacer and PAM sequence similarity based on the *Fusarium oxysporum* genome. Additionally, efforts were made to ensure that the sgRNA cleavage site is close to the 5’ region encoding the SIX9 gene of interest (GenBank: KX435015.1; Czislowski et al., 2018).

**Figure 1:**
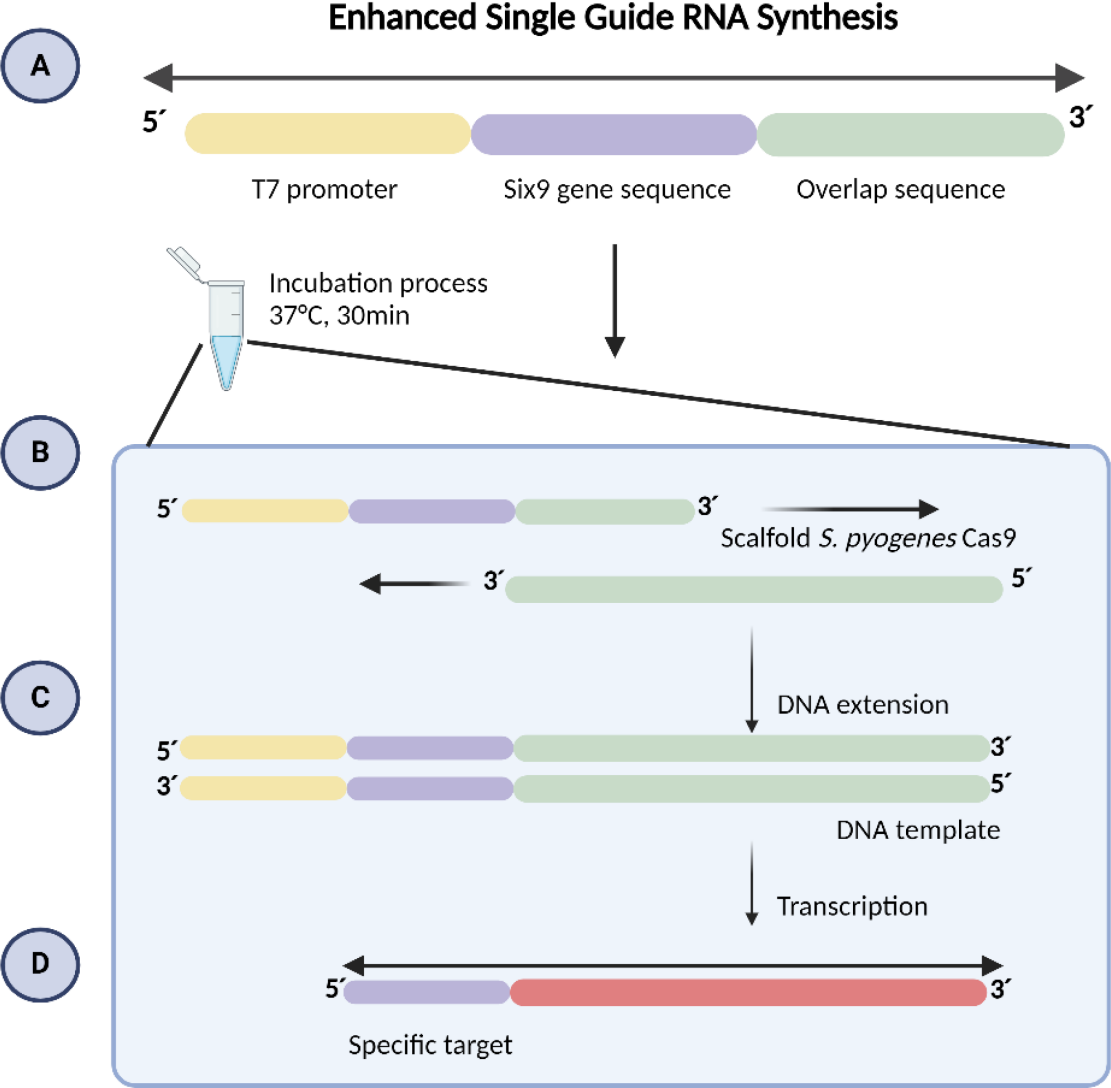
Guide RNA Synthesis. **(A)** The specific oligo contains the T7 promoter sequence, 20 nucleotides of the target sequence designed in silico, and a 14-nucleotide overlap sequence from S. pyogenes Cas9. **(B)** At 37°C, the two overlapping oligos align. **(C)** DNA polymerase extends both oligos from their 3’ ends, creating a double-stranded DNA template. **(D)** RNA polymerase recognizes the double-stranded T7 promoter DNA and initiates transcription.

The sgRNA consists of 20 nucleotides plus the PAM sequence (NGG; **Figure 1**), with the T7 promoter sequence (TTCTAATACGACTCACTATA) added to the 5’ region and a 14-nucleotide overlap sequence (GTTTTAGAGCTAGA) at the end of the 3’ region (**Table 1**).

**Table 1:** Sequences obtained from *in silico* design using Breaking-Cas software and the EnGen^®^ sgRNA Synthesis Kit, *S. pyogenes*.

SgRNA name	T7 Promoter Sequence	sgRNA	Overlap sequence
six9crispr1	TTCTAATACGACTCACTATA	GTCGCGCGTGCGTCTGAGTTA	GTTTTAGAGCTAGA
six9crispr2	TTCTAATACGACTCACTATA	GCAGATCTACGCTTTGGATTT	GTTTTAGAGCTAGA

For sgRNA synthesis, the EnGen^®^ sgRNA Synthesis *S. pyogenes* kit (NEB #E3322) was employed, following the manufacturer’s instructions. The reaction was incubated for 30 minutes at 37°C, after which the samples were transferred to ice. Additionally, a DNase treatment was performed by adding 30µl of ultrapure water and 2µl of DNase I, followed by incubation for an additional 15 minutes at 37°C. Subsequently, the samples were analyzed using a 2% agarose gel in 1X TAE buffer.

### 2.3 Preparation of plasmids

**Figure 2:**
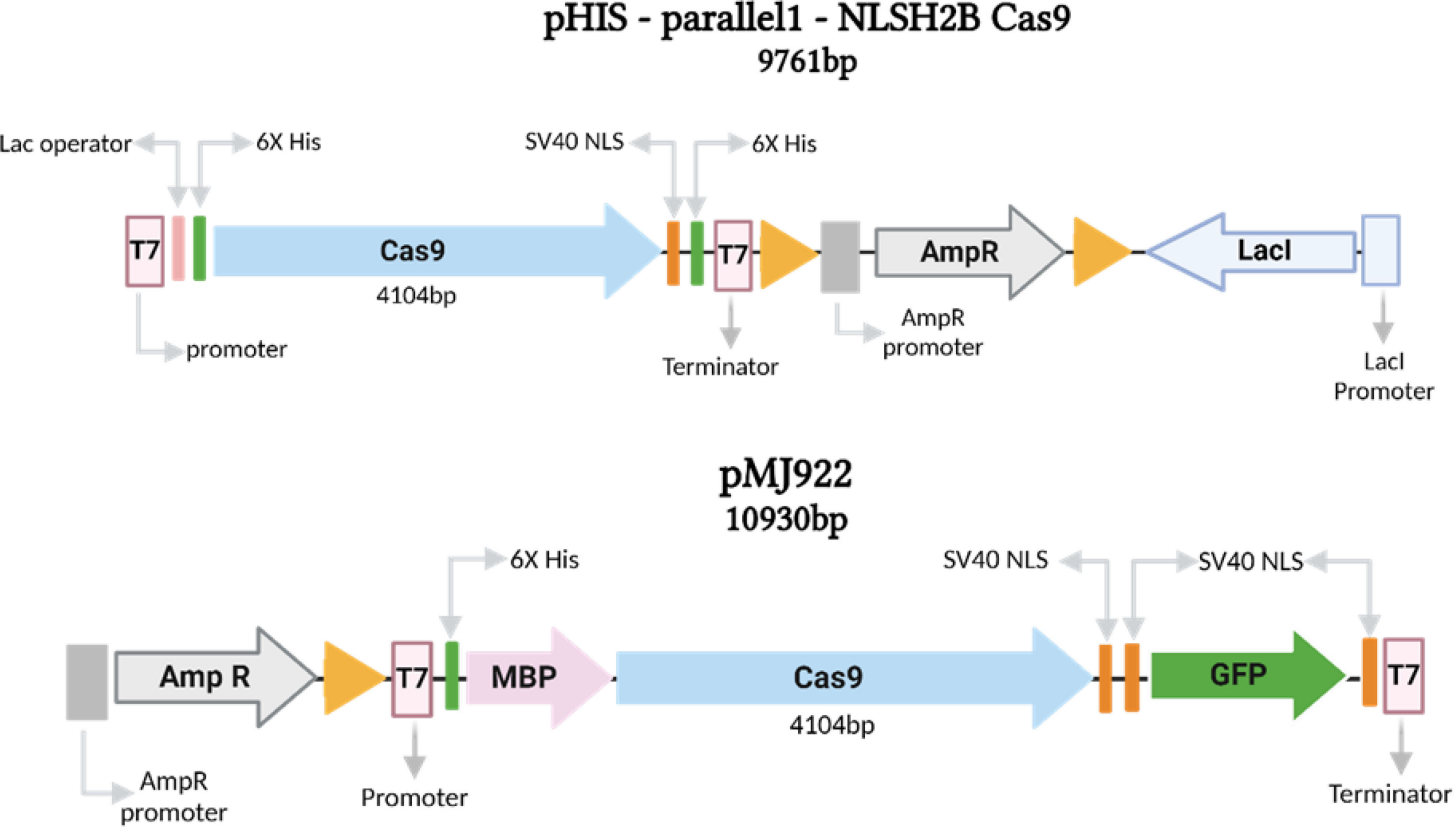
Linear diagram of the plasmids pHIS-parallel (9761bp) and pMJ922 (10930bp) used to produce the Cas9 protein. Both plasmids have the T7 promoter, nuclear localization signal (NLS) sites, and 6X Histidine tags to facilitate affinity purification. Finally, the plasmid pMJ922 fuses a codon-optimized SpCas9 nuclease from the CRISPR-Cas9 system of the bacterium *Streptococcus pyogenes*.

The pHis-parallel1-NLSH_2_BCas9 (pHIS) plasmid (9761 bp) and the pMJ922 (10930 bp) plasmid (**Figure 2**), both obtained from the Addgene repository, were utilized for this study (Burger et al., 2016; Wang et al., 2018).

The circular plasmid pHis-parallel1 is harbored within the *E. coli* Dh5alpha strain. The Cas9 coding sequence is fused with the nuclear localization signal (NLS) of histone H2B from *Fusarium oxysporum*. The Cas9 is an optimized coding sequence from *Streptococcus pyogenes* Cas9 (SpCas9), and coupled with the T7 promoter and terminator. The pMJ922 circular vector contains de Cas9 fused to the GFP reporter gene, a His6-MBP-TEV (N-terminal on the backbone) and HA-2xNLS-EGFP-NLS (C terminal on insert; **Figure 2**).

Petri dishes (PD) containing Luria Bertani (LB) culture medium (10 g Tryptone, 10 g NaCl, and 5 g yeast extract supplemented with 100 µg/mL of ampicillin per liter H_2_O) were used to isolate colonies. The PD were then incubated at 37°C for 24 hours. Following incubation, a single colony was selected and cultured in 5 mL of liquid LB medium supplemented with ampicillin (100 µg/mL) for 24 hours at 37°C.

Colony PCR was conducted to verify the presence of the target gene for the pHis-parallel1 plasmid, the DetCas9F (CGCGAGCGGATGAAGCGGAT) forward primer, and DetCas9R (CGCAGTTCCCA CGACTGCGT) reverse primer were used. The expected amplicon was 657 bp, while for the pMJ922 plasmid, the hSpCas9 FWD (AAGAAGGGCATCCTGCAGAC) forward primer and hSpCas9 REV (CTCGCTCTTGGCGATCATCT) reverse primer was employed expected amplicon of 879 bp.

The PCR protocol included an initial denaturation step at 94°C for 3 minutes, followed by 30 cycles of denaturation at 94°C for 30 seconds, annealing at 60°C for 30 seconds, and extension at 72°C for 1 minute, followed by a final extension step at 72°C for 5 minutes. Subsequently, plasmid extraction was performed using the PureLink™ Quick Plasmid Miniprep Kit (K210010).

**Figure 3:**
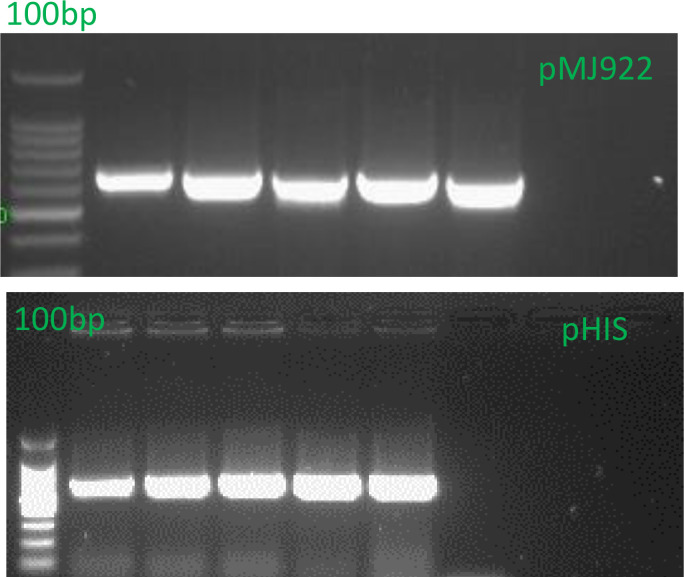
Colony PCR of *E. coli* was performed to confirm the presence of the Cas9 DNA region for the vector pMJ922. The expected band size was 879 bp, while the pHis-parallel1 insert was expected to yield a band of 657 bp. The PCR products were separated on a 1.2% agarose gel in 1X TAE buffer. The presence of the 879 bp band in the PCR results confirms the successful integration of the Cas9 DNA region into the vector, validating and confirming the correct insertion of the gene of interest. This result supports the transformation’s success and our experimental approach’s accuracy.

Subsequently, the obtained plasmids were cloned into *E. coli* BL21 Rosetta (Thermo Scientific, ECO114) expression bacteria. Plasmid presence was confirmed via colony PCR under the same conditions mentioned above (**Figure 3**). Finally, stocks were prepared with 20% glycerol and stored at -80°C.

### 2.4 Expression of Cas9 protein

From the glycerol stock at -80°C, initial PD for protein expression were prepared by streaking onto LB agar plates supplemented with 100 µg/mL ampicillin, covering the entire PD surface, and then left overnight at 37°C. The following day, using a sterile loop, the PD was scraped to collect the maximum number of bacteria and resuspended in 5 mL of LB medium with an antibiotic.

This bacterial suspension was subsequently added to 500 mL of Terrific Broth (TB) culture medium (24 g yeast extract, 20 g tryptone, and 4 mL glycerol per liter H_2_O), supplemented with 100 µg/mL ampicillin and 100 mL of sterile phosphate buffer (KH_2_PO_4_ 23.1g/L, K_2_HPO_4_ 125.4g/L). To ensure proper aeration, the assay was conducted in 2 L flasks.

The culture was then incubated at 37°C with agitation at 200 RPM for 4 hours or until the absorbance reached an O.D. of 0.6 Units at 600 nm. To prevent further bacterial proliferation, the culture was cooled to 4°C for approximately 30 minutes and then supplemented with 100 µg/mL ampicillin. At this point, an aliquot of the culture was taken for a miniprep to confirm the presence of the plasmid.

**Figure 4:**
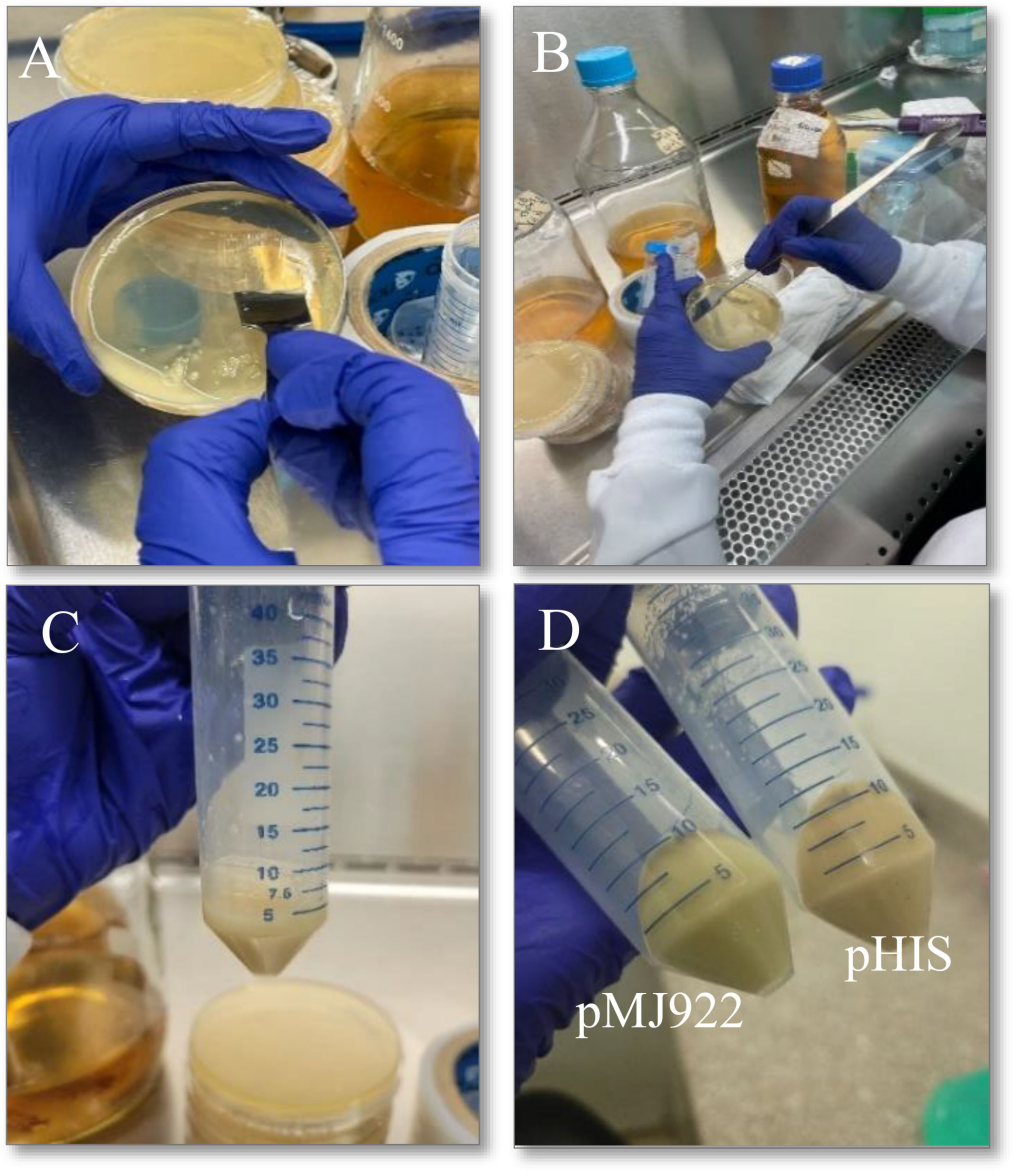
Process to obtain the *E*. *coli* culture containing the plasmids of interest. **(A, B)**: collection of *E*. *coli* in solid medium to start liquid incubation**, (C, D):** pelletized *E*. *coli* after incubation in liquid medium product of both plasmids, where pMJ922 contains the GFP reporter gene. A slight change in the color of the obtained product can already be noticed, and it was subsequently used in the cell lysis process.

Induction was performed with 200 µM Isopropyl β-D-thiogalactoside (IPTG; SIGMA, I6758), and the culture was maintained at 180 RPM for 16 hours at temperatures between 18 and 22°C. After this time, the bacteria was centrifuged at 3000 x g for 20 minutes at 4°C in 50 mL Falcon tubes. Once completed, the pellets obtained were weighed and stored at -80°C for subsequent use. At this stage of the process, the fluorescent activity of the green protein should be visible when using the plasmid pMJ922 (**Figure 4**).

### 2.5 Purification of Cas9 protein

The obtained pellets were weighed, and subsequently, bacterial lysis was performed. For this purpose, an IMAC A precipitation buffer (TRIS pH 7.5, NaCl 500 mM, MgCl_2_ 5 mM) was used at a 5:1 buffer-to-pellet ratio. The pellet underwent three freeze-thaw cycles at -80°C. Upon completion, the lysate was supplemented with a complete Roche protease inhibitor tablet (Cat: 04693132001), 1 mM DTT, 2 mM MgCl_2_, 2 µl RNase, and 2 µl DNase.

The lysate was kept on ice with gentle agitation for 30 minutes, ensuring minimal foaming. Subsequently, three sonication steps of 3 minutes each with 2-minute pauses in between were performed, keeping the samples under a cold chain. The lysate was centrifuged for 15 minutes at 16,000 x g at 4°C, and the supernatant was transferred to a new tube. A further 45-minute centrifugation step under the same conditions was carried out; if suspended debris persists, filtering the clarified solution is recommended.

#### 2.5.1 His-Tag Affinity Column Purification Process

In this step, HisPur™ Ni-NTA chromatography cartridges (Cat: Thermo Fisher Scientific, 88221) were used. Before the first use, the columns were washed with 10 volumes of ultrapure water and equilibrated with 10 volumes of IMAC A buffer. Subsequently, the clarified lysate obtained earlier was added through the columns, and an aliquot was taken for SDS-PAGE.

Next, the columns were washed with 25 volumes of IMAC A buffer, and the residual product was collected in case of protein loss in the following steps. The Cas9 protein was eluted using IMAC B buffer (50 mM TRIS pH 7.5, 250 mM NaCl, 5 mM MgCl_2_, 400 mM Imidazole), which contains a high concentration of imidazole to aid in the proper elution of the His-tagged protein through the IMAC columns.

**Figure 5:**
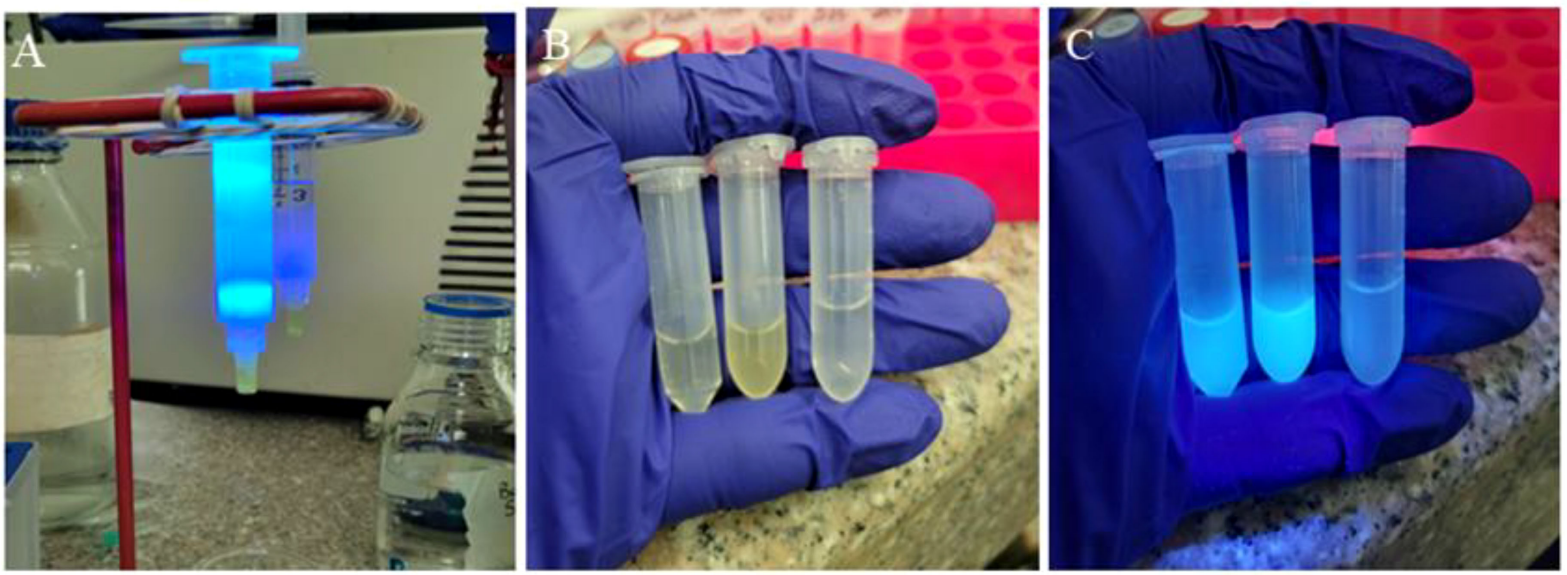
Purification of Cas9 protein. **(A)** Passage of the clarified lysate through the HisPur™ Ni-NTA chromatography column, **(B)** product obtained at the end of the elution under normal light, **(C)** product eluted with the pMJ922 plasmid containing the GFP under UV light.

Several fractions of approximately 500µl were collected (**Figure 5**); the column was washed with 20 volumes of IMAC B buffer, followed by 10 volumes of water, and finally stored in 20% ethanol. In the end, the presence of the protein was evaluated using the Bradford technique on an ELISA plate.

#### 2.5.2 Salt removal and buffer exchange

Additionally, salt removal was carried out using columns with Sephadex™ G-25 (Sigma-25150). The column was equilibrated with 20 mL of water, followed by 20 mL of LVA buffer (50 mM TRIS pH 7.5, 100 mM NaCl, and 5 mM MgCl_2_). Subsequently, 3 mL of the protein was loaded slowly; we also used 3 mL of LVA buffer for elution and added a reducing agent, which in this case was 1 mM DTT. The column was washed with the same buffer and stored in 20% ethanol adapted from (Fleitas et al., 2022).

#### 2.5.3 Cation exchange

As part of the purification process, a cation exchange was performed using columns with CM Sepharose (Cat: Sigma CL–6B-100). The columns were equilibrated with LVA buffer, and the protein was loaded onto the column. The unbound fraction was collected for analysis by SDS-PAGE (Fleitas et al., 2022).

Subsequently, a washing process was carried out using the same buffer, and LVB buffer (50 mM TRIS pH 7.5, 1M NaCl, and 5 mM MgCl_2_) was used for elution. Pierce Protein Concentrator PES columns (CAT. 88523) were used to concentrate the obtained protein. This involved a single centrifugation step for 5 minutes at 14,000g. The protein was quantified using a Nanodrop 2000 and finally stored at -80°C in different aliquots.

### 2.6 *In vitro* cleavage assay for targeted editing of the SIX9 gene

This study utilized recombinant Cas9 protein, purified using an optimized affinity and size-exclusion chromatography protocol. The cleavage assay was conducted in a reaction buffer optimized for Cas9 activity, consisting of HEPES (200 mM), NaCl (1 M), MgCl2 (50 mM), DTT (10 mM), and EDTA (1 mM), adjusted to pH 6.5. To ensure experimental rigor, the assay included three controls: a reaction with sgRNA targeting the SIX9 gene, a reaction without sgRNA, and another lacking both sgRNA and Cas9 as negative controls.

The ribonucleoprotein (RNP) complex was prepared by mixing 3 µg of Cas9 protein with sgRNA at a molar ratio of 3:1. Following the formation of the RNP complex, the final reaction mixture (20 µL) was assembled, comprising 17 µL of the Cas9-sgRNA complex, 1 µg of DNA template in 5 µL, and 5 µL of 10X reaction buffer. The reaction was incubated at 37°C for 1 hour in a thermal cycler. To terminate the enzymatic activity, 1 µL of proteinase K (20 mg/mL) was added, effectively chelating magnesium ions essential for Cas9 functionality.

The cleavage products were resolved by agarose gel electrophoresis, and the resulting DNA fragments were analyzed. Comparison of these fragments with positive and negative controls validated the specificity and efficiency of the Cas9-mediated cleavage.

## 3 Results and discussion

### 3.1 Optimized cloning and guide RNA design

To efficiently produce and purify the recombinant Cas9 protein, plasmids encoding the Cas9 sequence were cloned into the Rosetta strain of *Escherichia coli*. This strain is specifically optimized for expressing proteins requiring rare codons, often found in eukaryotic genes. The use of Rosetta ensures higher fidelity and yield during protein synthesis, making it a reliable choice for recombinant protein production (**Figure 3**).

In parallel, the guide RNAs (gRNAs) design was a step in the gene editing workflow. These gRNAs were meticulously designed to target the *SIX9* gene, implicated in *Fusarium* wilt resistance in bananas. The selection of gRNA sequences was guided by computational tools to ensure high specificity and minimal off-target effects, as these factors are essential for precise and efficient gene editing. The final sequences of the gRNAs employed in this study are detailed, providing a foundation for the subsequent editing process (**Table 1**).

### 3.2 Streamlined purification process for high-yield Cas9 protein

The purification of recombinant Cas9 protein is a critical step in ensuring its functional integrity for subsequent applications in gene editing. Given the complex nature of Cas9 and its pivotal role in CRISPR-based gene editing, achieving high yield and purity is paramount. To this end, a carefully optimized protocol was developed to isolate Cas9 from the *Escherichia coli* Rosetta strain, where it had been overexpressed.

The purification process began with cell lysis, utilizing mechanical and enzymatic methods to break open the bacterial cells and release the intracellular contents.

Following this, chromatographic techniques were employed to isolate the Cas9 protein. Initially, the crude lysate underwent affinity chromatography using a His-tag strategically included in the plasmid vector to facilitate easy capture of the recombinant protein (**Figure 5**). This step allowed for the initial enrichment of Cas9, removing a significant number of contaminating proteins.

### 3.3 Cas9 affinity purification process

**Figure 6:**
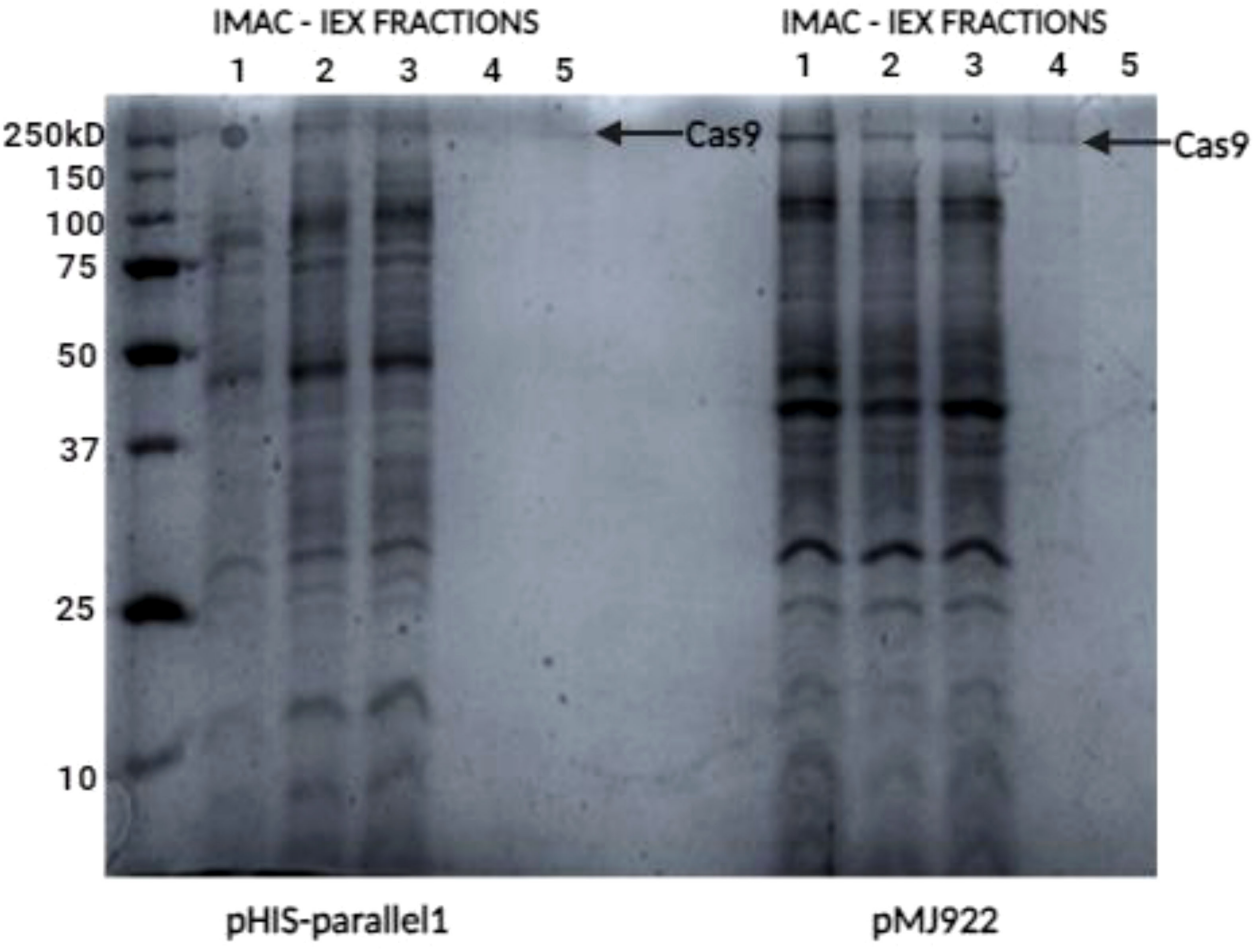
SDS-PAGE analysis for Cas9 protein detection. SDS-PAGE (12%) showed a faint band for the pHIS-parallel vector protein despite a 125 µg/mL concentration. In contrast, the pMJ922 vector displayed a clearer band under native conditions with a higher concentration of 225 µg/mL.

Subsequent purification steps included size-exclusion chromatography to refine the protein sample further, ensuring the removal of aggregates and misfolded proteins. This refinement process was critical for obtaining a highly pure and functional Cas9 protein. The final preparation underwent rigorous evaluation for purity using SDS-PAGE (**Figure 6**), while its functionality was confirmed through *in vitro* assays that demonstrated its ability to perform targeted DNA cleavage.

Several fractions were obtained from the purification process with the different Histidine columns (**Figure 5**). This process involved passing the samples through columns designed to bind proteins with histidine tags, allowing for selective purification. There was a significant difference in the concentration of both proteins in these fractions, which we attribute to the fact that the sample obtained from the pMJ922 vector was always fresh. Fresh samples typically yield higher protein concentrations due to reduced degradation and loss of activity.

To ensure accurate quantification, the obtained fractions were analyzed using the Nanodrop 2000, a spectrophotometer that measures the concentration of nucleic acids and proteins by assessing their absorbance at specific wavelengths. Additionally, we employed the Bradford technique to visually confirm the presence of the proteins, and this two-step verification process ensured the reliability and consistency of our protein purification results.

Further analysis was conducted using SDS-PAGE (12%) gel electrophoresis to evaluate the molecular weight and purity of the proteins. As shown in (**Figure 6**), wells loaded with 15 µL of the sample displayed varying results. Fractions derived from the pHIS-parallel vector showed a somewhat faint band corresponding to the expected size of the target protein despite achieving a quantification of 125 µg/mL. In contrast, fractions from the pMJ922 vector exhibited a more distinct and prominent band under native conditions, with a higher concentration of 225 µg/mL.

These results highlight the superior expression and stability of the protein purified from the pMJ922 vector, underscoring its effectiveness in recombinant protein production.

### 3.4 *In vitro* cleavage test of the SIX9 gene

**Figure 7:**
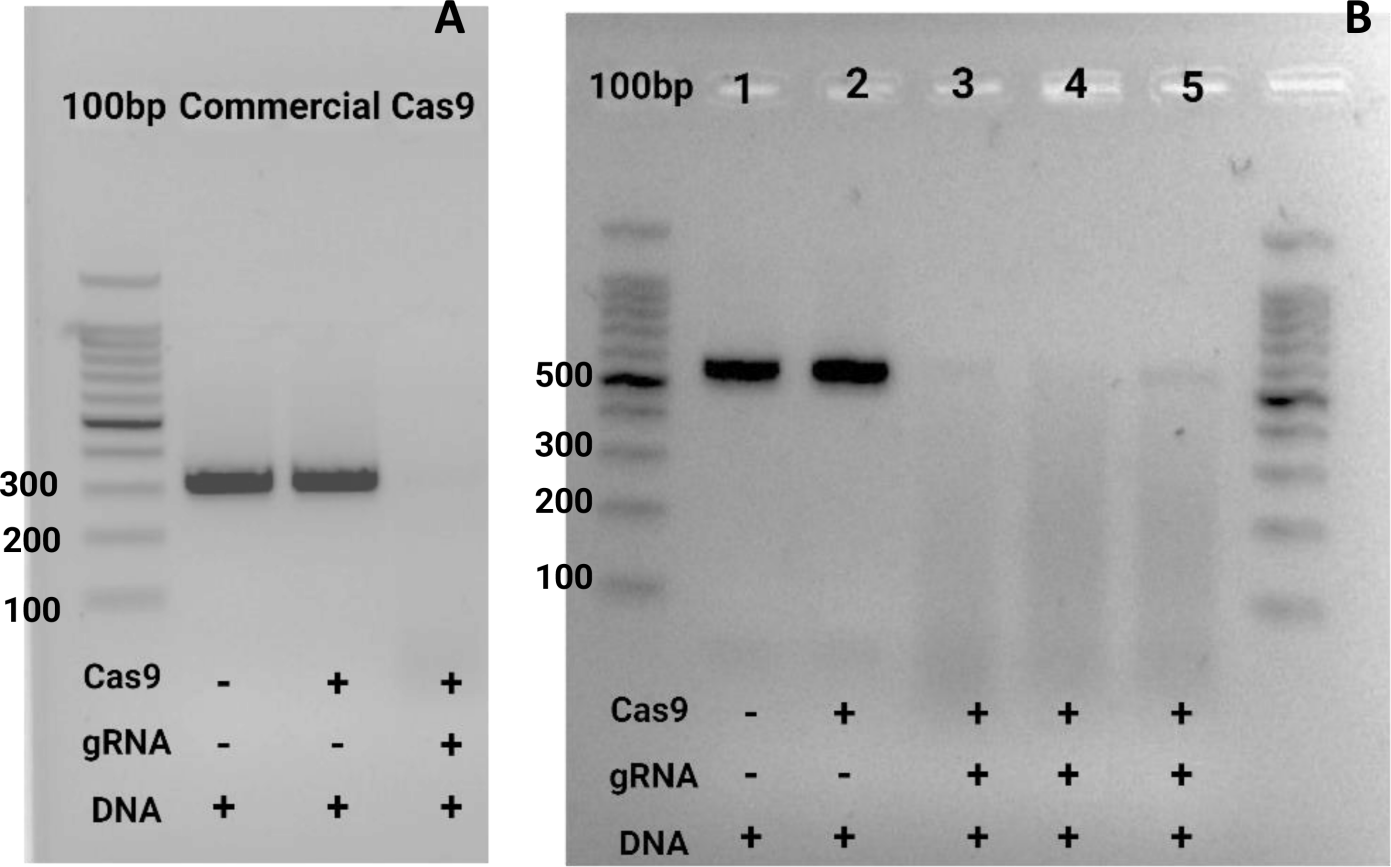
*In vitro* test for ribonucleoprotein (Cas9-gRNA) digestion of target amplicon. **(A)** The first well contained the 100 bp marker (Promega, G2101). The next two wells included negative controls with the DNA of interest (PCR product of target) without Cas9 or the guide. In the final well, all the samples were loaded. **(B)** In this image, two negative controls were used. The same gene of interest was tested, but the PCR product was amplified with the M13 primers to obtain a larger sequence of around 550 bp (from a pGEM vector with a cloned SIX9 PCR product). The third well contained the commercial Cas9 from Invitrogen, while wells 4 and 5 contained Cas9 obtained in the CIBE – ESPOL laboratory for all cases; guide 1 was used (**Table 1**). Both gels were run for 50 minutes at 90 volts in TAE buffer 1X 2.5% concentration.

After producing the Cas9 protein, we evaluated its efficacy compared to a commercial Cas9. This comparison aimed to determine whether our protocol could create a Cas9 protein with performance comparable to or exceeding the available commercial options. Initially, we focused on testing the commercial TrueCut Cas9 protein (Invitrogen, Cat # A36496) for this first round of testing (**Figure 7A**); we included two negative controls to ensure the reliability and accuracy of our results. These controls were crucial for identifying any potential background activity and for validating the specificity of the Cas9-mediated cleavage. In the subsequent phase of our evaluation (**Figure 7B**), we expanded the assay to include the commercial TrueCut Cas9 protein and the Cas9 proteins produced using our protocol. This allowed us to directly compare the performance of our in-house Cas9 with the established commercial standard. Including our Cas9 proteins in the assay was essential for assessing the practical application of our production process and identifying any necessary improvements or adjustments. The enzymatic reaction was carried out for 1 hour at 37°C, with deactivation for 10 minutes using proteinase K at the same temperature.

**Figure 8:**
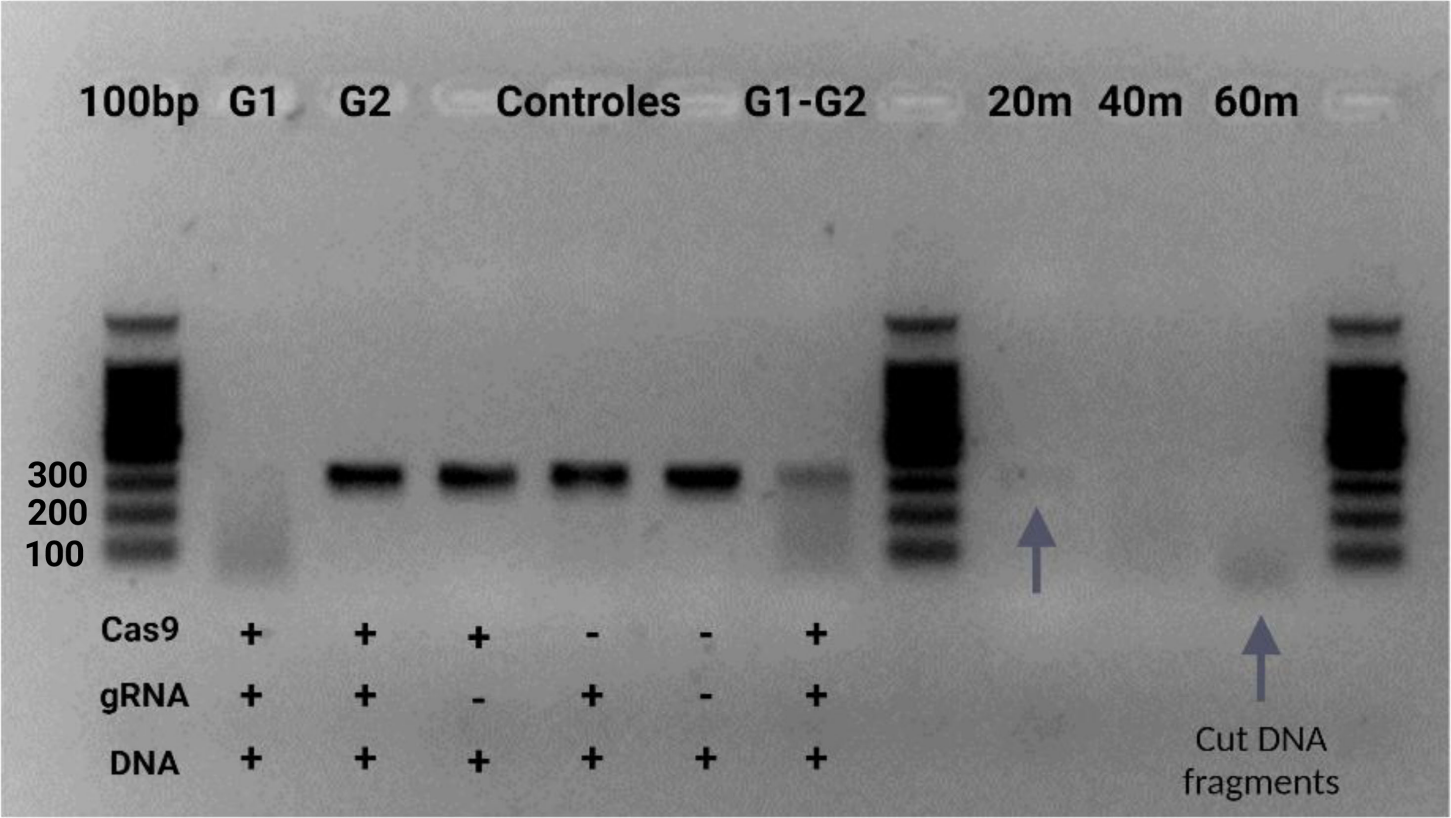
*In vitro* test for ribonucleoprotein (Cas9-gRNA) digestion of target amplicon using two gRNA. A 2.5% agarose gel was used with a 100bp marker. In wells 2 and 3, the enzymatic cleavage assay is shown, testing each of the designed guides. Wells 4-6 contain controls and a negative control. Guide one was used, and well 7 contains the cleavage assay with both guides. Wells 9-11 display the cleavage assays at different sampling times with the same guide.

In addition to these tests, an assay was conducted with the obtained protein, using the two RNA guides designed specifically for this purpose, individually and in combination. The results demonstrated consistency with previous findings using guide 1, as successful DNA cuts were achieved. Conversely, guide 2 was ineffective, as no DNA cuts were observed. The discrepancies could be attributed to some mismatch with the target sequence, particularly because it was a small sequence with several secondary structures in the gene of interest that might have hindered its function. Mismatches between the guide sequence and the target DNA are tolerated to some extent, especially in the region distal to the PAM sequence. These factors could explain why Guide 2 did not yield the desired results and why Guide 1 was preferred for subsequent experiments (Jinek et al., 2012). Samples were collected at various time intervals—20, 40, and 60 minutes—to monitor the progression of the enzymatic activity (**Figure 8**).

Initial signs of enzymatic cleavage were already apparent at the 20-minute mark; by 60 minutes, the target DNA fragment (SIX9, 316bp) was entirely disintegrated in the gel, reflecting the high efficiency of our Cas9 enzyme. The gel analysis revealed small DNA fragments accumulating in the lower regions, further confirming our system’s thorough breakdown of the DNA.

## 4 Discussion

The experimental design was developed to and rigorously evaluates Cas9-mediated DNA cleavage, incorporating appropriate controls, molecular markers, and optimized gel electrophoresis conditions. This methodology assesses the enzymatic activity of the Cas9 protein produced in-house at CIBE—ESPOL and enables a direct performance comparison with the commercially available TrueCut Cas9 enzyme. TrueCut was selected as the benchmark due to its established reliability and widespread use in genome editing applications (**Figure 7**).

Results demonstrated that the internally produced Cas9 exhibits high enzymatic activity, comparable to the commercial standard, thereby validating the efficacy of the production protocol and its potential for practical applications in gene editing. These findings underscore the robustness of the in-house enzyme and highlight its suitability for future research and biotechnological applications (Lee et al., 2022; Liu et al., 2022).

When used with guide one, the study found that our Cas9 protein successfully cleaved the target DNA fragment (SIX9, 316 bp) in 60 minutes, demonstrating high enzymatic activity comparable to the commercial standard. Using two RNA guides, tested individually and in combination, provides insight into their effectiveness in directing Cas9 to the target DNA sequence. These results are consistent with other studies, where the efficiency of Cas9 cleavage often varies depending on the RNA guide used (Liang et al., 2016; Tsakirpaloglou et al., 2023).

Temporal analysis of cleavage, with samples taken at 20, 40, and 60 minutes, further strengthens the study by showing the progressive nature of Cas9 activity (Mehravar et al., 2019). The complete degradation of the target DNA at 60 minutes highlights the high efficiency of our Cas9 enzyme, while the appearance of smaller DNA fragments confirms DNA degradation, indicating effective cleavage.

On the other hand, using negative controls (without Cas9 or guide) helped us confirm that any observed DNA cleavage is indeed the result of Cas9 activity and not due to other factors, such as contamination or spontaneous DNA degradation (Ly et al., 2023).

On the other hand, the variability in protein concentration observed in this study can be explained by the freshness of the sample, as mentioned in the text. Proteins in fresh samples tend to maintain their integrity and activity, leading to a higher concentration of purified product (Bornhorst and Falke, 2000; Rosano and Ceccarelli, 2014). Protein degradation in non-fresh samples can occur due to proteolytic activity or denaturation, resulting in a lower amount of functional protein for purification.

This study reveals that using RNP-CRISPR-Cas9 for genome editing is an efficient, accurate, and reproducible approach for gene manipulation, applicable to knock-out genes in *Fusarium oxysporum*.

## 5 Conclusions

The production of recombinant proteins is a crucial aspect of biotechnological research. This protocol facilitates the production of recombinant Cas9, enabling its use in various experimental settings and accelerating research in targeted gene editing, an area of significant relevance today. The SIX9 gene is recognized as a highly promising target due to its strong association with destructive diseases like *Fusarium* wilt in bananas, making it a critical focus for gene editing studies. *In vitro* digestion of specific genes using CRISPR/Cas9 in RNP format offers a straightforward and rapid method for pre-validating the CRISPR/Cas9 system before applying it to any cell type.

Future research could further explore additional parameters or process modifications to improve the performance of the internally produced Cas9, potentially creating a viable and cost-effective alternative to commercial options. Additionally, as a future outlook, there are plans to continue *in vivo* evaluations using transformation with protoplasts or electroporation, selecting the edited lines for potential greenhouse evaluation.

## Data Availability

The original contributions presented in the study are included in the article/supplementary material. Further inquiries can be directed to the corresponding author.

## References

[B1] An B. Hou X. Guo Y. Zhao S. Luo H. He C.. (2019). The effector SIX8 is required for virulence of Fusarium oxysporum f.sp. cubense tropical race 4 to Cavendish banana. Fungal Biol. 123 (5), 423–430. doi: 10.1016/j.funbio.2019.03.001 31053331

[B2] Bhattacharjee S. Bhowmick R. Kant L. Paul K. (2023). Strategic transgene-free approaches of CRISPR-based genome editing in plants. Mol. Genet. Genomics. 298 (3), 507–520. doi: 10.1007/s00438-023-01998-3 36840794 PMC9958309

[B3] Bornhorst J. A. Falke J. J. (2000). Purification of proteins using polyhistidine affinity tags. Methods Enzymol. 326, 245–254. doi: 10.1016/s0076-6879(00)26058-8 11036646 PMC2909483

[B4] Bragard C. Baptista P. Chatzivassiliou E. Di Serio F. Gonthier P. Jaques Miret J. A.. (2022). Pest categorisation of Fusarium oxysporum f. sp. cubense Tropical Race 4. EFSA J. 20 (1), 7092, 32 pp. doi: 10.2903/j.efsa.2022.7092 PMC8780018 35079290

[B5] Buddenhagen I. (2009). Understanding strain diversity in fusarium oxysporum f. sp. cubense and history of introduction of “tropical race 4“ to better manage banana production. Acta Hortic. 828, 193–204. doi: 10.17660/ActaHortic.2009.828.19

[B6] Burger A. Lindsay H. Felker A. Hess C. Anders C. Chiavacci E.. (2016). Maximizing mutagenesis with solubilized CRISPR-Cas9 ribonucleoprotein complexes. Dev. (Cambridge) 143 (11), 2025–2037. doi: 10.1242/dev.134809 27130213

[B7] Carvalhais L. C. Henderson J. Rincon-Florez V. A. O’Dwyer C. Czislowski E. Aitken E. A. B.. (2019). Molecular diagnostics of banana fusarium wilt targeting secreted-in-xylem genes. Front. Plant Sci. 10 (5), 1155–1171. doi: 10.3389/fpls.2019.00547 31214206 PMC6554419

[B8] Czislowski E. Fraser-Smith S. Zander M. O’Neill W. T. Meldrum R. A. Tran-Nguyen L. T. T.. (2018). Investigation of the diversity of effector genes in the banana pathogen, Fusarium oxysporum f. sp. cubense, reveals evidence of horizontal gene transfer. Mol. Plant Pathol. 19 (5), 1155–1171. doi: 10.1111/mpp.12594 28802020 PMC6638072

[B9] de Sain M. Rep M. (2015). The role of pathogen-secreted proteins in fungal vascular wilt diseases. Int. J. Mol. Sci. 16 (10), 23970–23993. doi: 10.3390/ijms161023970 26473835 PMC4632733

[B10] Dita M. Barquero M. Heck D. Mizubuti E. S. G. Staver C. P. (2018). Fusarium wilt of banana: Current knowledge on epidemiology and research needs toward sustainable disease management. Front. Plant Sci 9, 1468. doi: 10.3389/fpls.2018.01468 30405651 PMC6202804

[B11] Douches D. S. Nadakuduti S. S. Enciso F. Carpintero N. M. (2020). Genome editing for potato (Solanum tuberosum L.)–current status and future prospects. IOP Conf Ser. Earth Environ. Sci. 482, 012004. doi: 10.1088/1755-1315/482/1/012004

[B12] Evizal R. Prasmatiwi F. E. (2024). Bananas Intercropping Effects on Cocoa Yield and Land Productivity. IOP Conference Series: Earth and Environmental Science 1417 (1), 012004. doi: 10.1088/1755-1315/1417/1/012004

[B13] FAO (2023). Banana market review: Preliminary results 2023 (Food and Agriculture Organization of the United Nations). https://openknowledge.fao.org/items/7903534c-c733-482d-b05c-37ad8245afa2

[B14] Feng S. Wang Z. Li A. Xie X. Liu J. Li S.. (2022). Strategies for high-efficiency mutation using the CRISPR/cas system. Front. Cell Dev. Biol 9, 803252. doi: 10.3389/fcell.2021.803252 35198566 PMC8860194

[B15] Fleitas A. L. Señorale M. Vidal S. (2022). A robust expression and purification method for production of spCas9-GFP-MBP fusion protein for *in vitro* applications. Methods Protoc. 5 (3), 44. doi: 10.3390/mps5030044 35736545 PMC9228339

[B16] Gawehns F. Ma L. Bruning O. Houterman P. M. Boeren S. Cornelissen B. J. C.. (2015). The effector repertoire of Fusarium oxysporum determines the tomato xylem proteome composition following infection. Front. Plant Sci. 6. doi: 10.3389/fpls.2015.00967 PMC4631825 26583031

[B17] Gong Z. Cheng M. Botella J. R. (2021). Non-GM genome editing approaches in crops. Front. Genome Ed. 3, 817279. doi: 10.3389/fgeed.2021.817279 34977860 PMC8715957

[B18] González I. Arias Y. Peteira B. (2012). Artículo reseña ASPECTOS GENERALES de la INTERACCIÓN Fusarium oxysporum f. sp. lycopersici-TOMATE, Rev. Protección Veg. 27 (1), 1–7. Available online at: http://scielo.sld.cu/scielo.php?script=sci_arttext&pid=S101027522012000100001&lng=es&tlng=es.

[B19] Houterman P. M. Speijer D. Dekker H. L. De Koster C. G. Cornelissen B. J. C. Rep M. (2007). The mixed xylem sap proteome of Fusarium oxysporum-infected tomato plants: Short communication. Mol. Plant Pathol. 8 (2), 215–221. doi: 10.1111/j.1364-3703.2007.00384.x 20507493

[B20] Huang Y. Y. Zhang X. Y. Zhu P. Ji L. (2022). Development of clustered regularly interspaced short palindromic repeats/CRISPR-associated technology for potential clinical applications. World J. Clin. cases 10 (18), 5934–5945. doi: 10.12998/wjcc.v10.i18.5934 35949837 PMC9254185

[B21] Izquierdo-García L. F. Carmona S. L. Zuluaga P. Rodríguez G. Dita M. Betancourt M.. (2021). Efficacy of disinfectants against fusarium oxysporum F. Sp. cubense tropical race 4 isolated from La Guajira, Colombia. J. Fungi 7 (4), 297. doi: 10.3390/jof7040297 PMC8071173 33920770

[B22] Jangir P. Mehra N. Sharma K. Singh N. Rani M. Kapoor R. (2021). Secreted in xylem genes: drivers of host adaptation in fusarium oxysporum. Front. Plant Sci. 12, 628611. doi: 10.3389/fpls.2021.628611 33968096 PMC8101498

[B23] Jobe T. O. Urner M. Ulloa M. Broders K. Hutmacher R. B. Ellis M. L. (2024). Secreted in Xylem (SIX) Gene SIX9 Is Highly Conserved in Fusarium oxysporum f. sp. vasinfectum Race 4 Isolates from Cotton in the United States. PhytoFrontiersTM. doi: https://doi.org/10.1094/phytofr-11-23-0143-sc

[B24] Keeratiburana T. Siangwengwang N. Somphunga W. Fu T. Blennow A. (2024). Ultrasound-assisted annealing treatment to improve physicochemical and digestive properties of banana flour. Journal of the Science of Food and Agriculture 104 (11), 6640–6648. doi: 10.1002/JSFA.13488 38523359

[B25] Kumari P. Gaur S. S. Tiwari R. K. (2023). Banana and its by-products: A comprehensive review on its nutritional composition and pharmacological benefits. In eFood 4 (5). doi: https://doi.org/10.1002/efd2.110

[B26] Laforest L. C. Nadakuduti S. S. (2022). Advances in delivery mechanisms of CRISPR gene-editing reagents in plants. Front. Genome Ed 4, 830178. doi: 10.3389/fgeed.2022.830178 35141701 PMC8819002

[B27] Lee N. Park J. Kim J. E. Shin J. Y. Min K. Son H. (2022). Genome editing using preassembled CRISPRCas9 ribonucleoprotein complexes in Fusarium graminearum. PLoS One 17 (6), e0268855. doi: 10.1371/journal.pone.0268855 35657788 PMC9165886

[B28] Liang G. Zhang H. Lou D. Yu D. (2016). Selection of highly efficient sgRNAs for CRISPR/Cas9-based plant genome editing. Sci. Rep. 6, 21451. doi: 10.1038/srep21451 26891616 PMC4759811

[B29] Lievens B. Houterman P. M. Rep M. (2009). Effector gene screening allows unambiguous identification of Fusarium oxysporum f. sp. lycopersici races and discrimination from other formae speciales. FEMS Microbiol. Lett. 300 (2), 201–215. doi: 10.1111/j.1574-6968.2009.01783.x 19799634

[B30] Lino C. A. Harper J. C. Carney J. P. Timlin J. A. (2018). Delivering crispr: A review of the challenges and approaches. Drug Delivery. doi: 10.1080/10717544.2018.1474964 PMC6058482 29801422

[B31] Liu Y. Andersson M. Granell A. Cardi T. Hofvander P. Nicolia A. (2022). Establishment of a DNA-free genome editing and protoplast regeneration method in cultivated tomato (Solanum lycopersicum). Plant Cell Rep. 41 (9), 1843–1852. doi: 10.1007/s00299-022-02893-8 35773498 PMC9395478

[B32] Loeillet D. C. (2023). Close-up. Banana volumes review. FruiTrop 293, 40–49. www.fruitrop.com.

[B33] López-Zapata S. P. Castaño-Zapata J. (2019). Manejo integrado del mal de Panamá [Fusarium oxysporum Schlechtend.: Fr. sp. cubense (E.F. SM.) W.C. Snyder & H.N. Hansen]: una revisión. Rev. U.D.C.A Actualidad Divulgación Científica 22 (2), e1240. doi: 10.31910/rudca.v22.n2.2019.1240

[B34] Ly D. N. P. Iqbal S. Fosu-Nyarko J. Milroy S. Jones M. G. K. (2023). Multiplex CRISPR-cas9 gene-editing can deliver potato cultivars with reduced browning and acrylamide. Plants 12 (2), 379. doi: 10.3390/plants12020379 36679094 PMC9864857

[B35] Magdama F. Monserrate-Maggi L. Serrano L. Onofre J. G. Jiménez-Gasco M. D. M. (2020). Genetic diversity of fusarium oxysporum f. Sp. cubense, the fusarium wilt pathogen of banana, in Ecuador. Plants 9 (9), 1133. doi: 10.3390/plants9091133 32882937 PMC7570379

[B36] Magdama F. Monserrate-Maggi L. Serrano L. Sosa D. Geiser D. M. Jiménez-Gasco M.. (2019). Comparative analysis uncovers the limitations of current molecular detection methods for Fusarium oxysporum f. Sp. Cubense Race 4 strains. PLoS One 14 (9), e0222727. doi: 10.1371/journal.pone.0222727 31545825 PMC6756539

[B37] Marraffini L. A. (2016). The CRISPR-Cas system of *Streptococcus pyogenes*: function and applications. In Ferretti J. J. (Eds.) Streptococcus pyogenes: Basic Biology to Clinical Manifestations. University of Oklahoma Health Sciences Center. 27077169

[B38] Martínez G. Olivares B. O. Rey J. C. Rojas J. Cardenas J. Muentes C.. (2023). The advance of fusarium wilt tropical race 4 in musaceae of latin america and the caribbean: current situation. Pathogens 12 (2), 277. doi: 10.3390/pathogens12020277 36839549 PMC9963102

[B39] Martínez-Solórzano G. E. Rey-Brina J. C. Pargas-Pichardo R. E. Enrique-Manzanilla E. (2020). Marchitez por Fusarium raza tropical 4: Estado actual y presencia en el continente americano 1 Fusarium wilt by tropical race 4: Current status and presence in the American continent. Agronomía Mesoamericana 31 (1), 259–276. doi: 10.15517/am.v31i1.37925

[B40] Maymon M. Sela N. Shpatz U. Galpaz N. Freeman S. (2020). The origin and current situation of Fusarium oxysporum f. sp. cubense tropical race 4 in Israel and the Middle East. Sci. Rep. 10, 1590. doi: 10.1038/s41598-020-58378-9 32005853 PMC6994609

[B41] Mehravar M. Shirazi A. Mehrazar M. M. Nazari M. (2019). *In vitro* pre-validation of gene editing by CRISPR/Cas9 ribonucleoprotein. Avicenna J. Med. Biotechnol. 11 (3), 259–263. 31380000 PMC6626505

[B42] Meiliana A. Dewi N. M. Wijaya A. (2017). Genome editing with CRISPR-Cas9 systems: Basic research and clinical applications. Indonesian Biomed. J. 9, 1–16. doi: 10.18585/inabj.v9i1.272

[B43] Michielse C. B. Rep M. (2009). Pathogen profile update: Fusarium oxysporum. Mol. Plant Pathol. 10 (3), 311–324. doi: 10.1111/j.1364-3703.2009.00538.x 19400835 PMC6640313

[B44] Munhoz T. Vargas J. Teixeira L. Staver C. Dita M. (2024). Fusarium Tropical Race 4 in Latin America and the Caribbean: status and global research advances towards disease management. Front. Plant Sci. 15. doi: 10.3389/fpls.2024.1397617 PMC11286425 39081528

[B45] Nadakuduti S. S. Enciso-Rodríguez F. (2021). Advances in genome editing with CRISPR systems and transformation technologies for plant DNA manipulation. Front. Plant Sci. 11, 637159. doi: 10.3389/fpls.2020.637159 33519884 PMC7840963

[B46] O’Neill W. T. Henderson J. Pattemore J. A. O’Dwyer C. Perry S. Beasley D. R.. (2016). Detection of Fusarium oxysporum f. sp. cubense tropical race 4 strain in northern Queensland. Australas. Plant Dis. Notes 11, 33. doi: 10.1007/s13314-016-0218-1

[B47] Oliveros J. C. Franch M. Tabas-Madrid D. San-León D. Montoliu L. Cubas P.. (2016). Breaking-Cas-interactive design of guide RNAs for CRISPR-Cas experiments for ENSEMBL genomes. Nucleic Acids Res. 44 (W1), W267–W271. doi: 10.1093/NAR/GKW407 27166368 PMC4987939

[B48] Ordonez N. Seidl M. F. Waalwijk C. Drenth A. Kilian A. Thomma B. P. H. J.. (2015). Worse comes to worst: bananas and Panama disease—When plant and pathogen clones meet. PLoS Pathog. 11 (11), e1005197. doi: 10.1371/journal.ppat.1005197 26584184 PMC4652896

[B49] Peterson D. Barone P. Lenderts B. Schwartz C. Feigenbutz L. St. Clair G.. (2021). Advances in Agrobacterium transformation and vector design result in high-frequency targeted gene insertion in maize. Plant Biotechnol. J. 19 (10), 2000–2010. doi: 10.1111/pbi.13613 33934470 PMC8486252

[B50] Ploetz R. C. (2015). Management of Fusarium wilt of banana: A review with special reference to tropical race 4. Crop Prot. 73, 7–15. doi: 10.1016/j.cropro.2015.01.007

[B51] Redkar A. Sabale M. Schudoma C. Zechmann B. Gupta Y. K. López-Berges M. S.. (2022). Conserved secreted effectors contribute to endophytic growth and multihost plant compatibility in a vascular wilt fungus. Plant Cell 34 (9), 3214–3232. doi: 10.1093/plcell/koac174 35689625 PMC9421472

[B52] Rep M. van der Does H. C. Meijer M. Van Wijk R. Houterman P. M. Dekker H. L.. (2004). A small, cysteine-rich protein secreted by Fusarium oxysporum during colonization of xylem vessels is required for I-3-mediated resistance in tomato. Mol. Microbiol. 53 (5), 1373–1383. doi: 10.1111/j.1365-2958.2004.04177.x 15387816

[B53] Rosano G. L. Ceccarelli E. A. (2014). Recombinant protein expression in Escherichia coli: Advances and challenges. Front. Microbiol. 5, 172. doi: 10.3389/fmicb.2014.00172 24860555 PMC4029002

[B54] Sandhya D. Jogam P. Allini V. R. Abbagani S. Alok A. (2020). The present and potential future methods for delivering CRISPR/Cas9 components in plants. J. Genet. Eng. Biotechnol. 18 (1), 25. doi: 10.1186/s43141-020-00036-8 32638190 PMC7340682

[B55] Staver C. Pemsl D. E. Scheerer L. Perez Vicente L. Dita M. (2020). Ex Ante Assessment of Returns on Research Investments to Address the Impact of Fusarium Wilt Tropical Race 4 on Global Banana Production. Frontiers in Plant Science 11, 804. doi: https://doi.org/10.3389/fpls.2020.00844 32733497 PMC7357546

[B56] Tsakirpaloglou N. Septiningsih E. M. Thomson M. J. (2023). Guidelines for performing CRISPR/cas9 genome editing for gene validation and trait improvement in crops. Plants. 12 (20), 3564. doi: 10.3390/plants12203564 37896028 PMC10610170

[B57] Villao-Uzho L. Chávez-Navarrete T. Pacheco-Coello R. Sánchez-Timm E. Santos-Ordóñez E. (2023). Plant promoters: their identification, characterization, and role in gene regulation. Genes (Basel). 14 (6), 1226. doi: 10.3390/genes14061226 37372407 PMC10298551

[B58] Wang Q. Cobine P. A. Coleman J. J. (2018). Efficient genome editing in Fusarium oxysporum based on CRISPR/Cas9 ribonucleoprotein complexes. Fungal Genet. Biol. 117, 21–29. doi: 10.1016/j.fgb.2018.05.003 29763675 PMC6480338

[B59] Wei J. Li Y. (2023). CRISPR-based gene editing technology and its application in microbial engineering. Eng. Microbiol. 3 (4), 100101. doi: 10.1016/j.engmic.2023.100101 39628916 PMC11610974

[B60] Woo J. W. Kim J. Kwon S. Corvalán C. Cho S. W. Kim H.. (2015). DNA-free genome editing in plants with preassembled CRISPR-Cas9 ribonucleoproteins. Nat. Biotechnol. 33, 1162–1164. doi: 10.1038/nbt.3389 26479191

[B61] Wu X. Kriz A. J. Sharp P. A. (2014). Target specificity of the CRISPR-Cas9 system. Quantitative Biol. 2 (2), 59–70. doi: 10.1007/s40484-014-0030-x PMC4338555 25722925

[B62] Yu D. S. Outram M. A. Smith A. McCombe C. L. Khambalkar P. B.. (2024). The structural repertoire of Fusarium oxysporum f. sp. lycopersici effectors revealed by experimental and computational studies. ELife 12, RP89280. doi: https://doi.org/10.7554/eLife.89280 38411527 PMC10942635

[B63] Zhang S. Shen J. Li D. Cheng Y. (2020). Strategies in the delivery of Cas9 ribonucleoprotein for CRISPR/Cas9 genome editing. Theranostics. 11 (2), 614–648. doi: 10.7150/thno.47007 PMC7738854 33391496

